# Effect of Environment

**Published:** 1893-06

**Authors:** 


### EFFECT OF ENVIRONMENT.

Children are peculiarly susceptible to the beauty or otherwise of their surroundings. They may not be able to voice it—may not be conscious of it, even, but it has none the less a potent influence on their behavior. "I used to notice," said an observing person once, "in a family which I visited quite frequently that when my call was confined to a chat in the library, a lovely ennobling room, full of books and sunshine, if the children were visible at all they were exceedingly mannerly and charming, while on the occasions when I would go down informally to the home luncheon or dinner, their behavior was quite different. The room was dark and sunless, and the belongings good, but all freshness worn off. I finally attributed the change in the children's conduct to their different environment."

